# The Modern Treatment of Neuralgia

**Published:** 1888-12

**Authors:** Landon Carter Gray

**Affiliations:** Professor of Mental and Nervous Diseases in the New York Polyclinic


### THE MODERN TREATMENT OF NEURALGIA.

By LANDON CARTER GRAY, M. D.,
Professor of Mental and Nervous Diseases in the New York Polyclinic.

The modern study of nervous diseases has modified many of the old ideas about the nature of neuralgia, and added many new details of great value in the treatment For example, the time-honored saying that "neuralgia is the outcry of an impoverished blood," expressed only the incomplete knowledge of the older physicians of a time when the vascular system was held to be the cause of all abnormal phenomena of the human body, and gives emphasis to what the later developments of science have shown to be only one of the many causes of neuralgia, and that, too, a very subsidiary one.

Before considering more immediately the subject of my paper, I desire to lay great emphasis upon the fact that distinction should always be made between neuralgia and neuritis. I scarcely need to remind your learned body that a peripheral nerve is made up of the axis-cylinder, a continuous nitrogenized body running from the central nervous organs to the sensory structures of the periphery; of a fatty sheath surrounding the axis-cylinder, and known as the medullary sheath; and, finally, of a delicate, transparent, connective-tissue sheath, enveloping both medullary layer and axis-cylinder like a glove, and designated as the sheath of Schwann. A neuritis; or inflammation of this peripheral nerve in these three structures—axis-cylinder, medullary layer, sheath of Schwann—causes structural changes which are visible under the microscope. These changes are manifold, and differ in degree according to the severity of the inflammation. In the lighter forms of neuritis the delicate connective-tissue sheath may alone be involved, so that its structure becomes somewhat thickened, and it loses its translucent appearance, becoming more and more opaque; the little cellular bodies, the nuclei, increase in number and size, and take up a greater quantity of the coloring matter which may be used, such as carmine or hematoxylin, whereas in health they are very sparse and not easily colorable. In the types of neuritis that are somewhat more severe the fatty layer surrounding the axis-cylinder, the medullary layer, becomes implicated, and breaks up more or less into its constituent fat, so that it loses its even and continuous appearance, or is dissolved into irregular fatty chunks, or even into actual fat globules. Finally, in the most severe types, the axis-cylinder becomes altered in shape, or becomes partially or entirely disintegrated. The clinical symptoms indicative of these molecular changes, which are known by the name of neuritis, are:

Pain which is steadily fixed in the distribution of the particular nerve affected.

In the severer forms, a swelling of the region to which the affected nerves are distributed, the swelling being accompanied by a glossy and hot skin.

Often by actual motor paralysis of the muscles to which the affected nerve is distributed.

Neuralgia on the other hand, is a functional affection, which is unacc mpanied by any sign of moleclular alteration discoverable in the nerve affected; and the clinical symptom which denotes this functional affection is pain alone, which may be mainly located in one nerve, but which almost invariably jumps at intervals from this nerve to others in the neighborhood, or to symmetrical ones upon the opposite side of the body.

It may happen of course, that a long continued neuralgia may lead to neuritis; or it may happen on the other hand, that a neuritis of the sub-acute or chronic type may begin with symptoms of a neuralgic character. But the differentiation can easily be made as the disease progresses.

Although our knowledge of the causation of neuralgia still lacks precision, we are yet cognizant of a number of etiological factors for whose possible existence in any given case we should carefully examine. We should always search for a history of malaria, and not only for a history of actually existing malaria, but for a history of preceding malaria. If an individual lives in a malarious district, and has distinct malarial attacks, with typical chill, fever and sweating, we are not likely to be thrown off our guard. But it should also be borne in mind, as is not generally known, that an individual may have been subject to malaria in the past, not have any rise in the temperature or chill or sweating in the present, and yet have a distinct periodical neuralgia left as a relic of the precedent malarial condition. For instance, many such cases of neuralgia will come on at regular periods, which are just exact in their arrival as were the full-fledged malarial attacks precedent to them, often in the distant past. All the treatment in the world addressed to this form of neuralgia between these periodical periods is worse than useless, inasmuch as it often aggravates the suffering; and yet they can usually be promptly cured when medicine is directed to tbem at the proper time. Then, too, the condition known as lithæia is often the cause of neuralgia—*i. e.*, a condition in which the tongue is coated, the bowels are somewhat constipated, the patient complains of vertigo, various tingling, furry and abnormal sensations in the extremities. Lithæmia is a very frequent sequel of prolonged malarial infection, is often seen in malarial districts even when no distinct symptoms of malaria are present, and is a frequent phenomenon in individuals who are personally or hereditarily of gouty or rheumatic tendencies. Atmospheric variations, which are so frequent in this American country of ours, constitute another fertile source of neuralgia. Uncorrected errors of ocular refraction occasionally set up neuralgia, such as astigmatism, hypermetropia, and myopia; although these causes are not nearly so frequent as certain of our writers would make them out to be.

In any given case, therefore, careful regard should be had to the distinction between neuralgia and neuritis, to a possible malarial infection, to a possible post malarial periodicity, to a possible lithæmia, to a possible exposure to great atmospheric variations, to possible errors of ocular refraction.

If malaria be present, it should be broken up vigorously by means of the usual treatment upon which I need not dwell, and in most cases the neuralgia will then disappear. If there be a post-malarial periodicity, the treatment should be addressed to the periodical attacks alone. Some six to twelve hours before the attack is expected, quinine sulph., gr. v–xx—or even xxx should be given conjoined with morphiæ acetatis, gr. 1/8–1-6, which latter should be given hypodermically, if that be possible. If the individual be strong and robust, the breaking up of the periodicity will usually cure the neuralgia, and no further treatment will be needed. If the individual be not robust, it may be necessary to follow the treatment, after the periodicity has been broken up, by means of drugs which have been found useful in p st-malarial sequelæ. The best of these are arsenic, best given in Fow'er's solution, gtt. ij–iij, three times a day, and iron, preferable the ticture of the muriate, gtt. xx–xxx, three times a day.

If lithæmia be present, the patient should be put upon a mineral acid and laxatives. The best acid for this purpose is the dilute nitro-muriatic, of which 20 drops should be given three times daily, before meals, in a wineglass of water, and this continued for two or three weeks. Laxatives should be used gently, so as to cause easy and somewhat liquid movements, but never to purge or to act with watery transudation. If the lithæmia be post-malarial, calomel, 1 to 2 grains, given at bed time, and followed in the morning, if necessary, with a small dose of a saline, may often be used with great benefit, provided that the patient be strong and hearty, but it should always be borne in mind that calomel may act very unfavorably upon the neuralgia if the patient be weak or debilitated. In any event, the calomel should not be used more than once, or at long intervals. Usually the best preparation, if I may judge from a considerable experience, is the fluid extract of cascara sagrada. The great value of this drug is that it can be increased or diminished, according to the idiosyncracy of the patient, with absolute impunity, so that one patient may get all the desired effects from 10 drops once daily, whilst another may have to take one ounce three times daily for the same purpose. My rule is to commence with one-drachm doses three times a day, telling the patient that I want him only to have one or two easy liquid stools in the course of the day, and then let them regulate the dose for themselves. After this laxative and mineral acid treatment has continued for ten days or two weeks, it may be stopped, and a bitter tonic should be given in place of the acid and the laxative should only be used accordingly as necessity may arise.

If the neuralgia has been plainly caused by some great atmospheric variation, or by a series of them, the patient should, if possible, be removed to a more equable climate, when it is seen that other treatment has failed.

The errors of refraction should be carefully corrected by means of glasses that should be adapted to the needs of the patient by some competent oculist, and not by the harum-scarum methods of the ordinary shop-keeping optician.

The neuralgia will cease in a certain proportion of cases upon the removal of the above causative factors. Should it not do so, however—and the neuralgic habit often keeps up after a perfectly adequate cause for it has been removed—or should it appear without any of these above-mentioned causes, our treatment must be directed to the malady itself, and then it is that we must make distinction between neuralgia of the tregiminus, of the sciatic, and of the nerves of the trunk and extremities, as each has peculiarities of its own.

In trigeminal neuralgia, defective teeth should always be looked for and carefully attended to; and in this form electricity seems to be of little or no use.

In sciatic neuralgia, the patient should be put absolutely to bed for a week or two at the commencement of the treatment, for we are dealing with a long and large nerve which is irritated by every movement of the extremity, and here we must carefully search, by a rectal examination, for a possible intra-pelvic tumor.

If these clinical differences be borne in mind, the treatment of these different forms may be the same. It may be laid down as an axiom that pain should be promptly checked until we have had time to directly treat the disease. In every case the affected nerve should be placed absolutely at rest. If the lower extremity or the trunk be affected, the patient should be put into bed and made to quietly stay there. It is utterly impossible to otherwise keep a leg or the abdominal muscles at rest. If an upper extremity be affected, it should be carried in a sling, and not used. The most effective drugs for the prompt relief of pain are antipyrin, antifebrin and the alkaloids of opium. In a large proportion of cases antipyrin and antifebrin will do all that morphia can do. Antipyrin should be given 5-grain doses every hour until the pain is relieved, or a maximum dosage of 40 grains has been attained in twenty-four hours; but in using this drug, the heart should always be carefully examined, so as to avoid administering it in organic valvular lesions, and it should also be borne in mind that it is very depressing in its action in many cases. For this latter reason antifebrin has of late come into use, because it does not depress; yet, I am sorry to say, antifebrin has not anything like the pain-relieving properties of antipyrin, although it is well to make trial of it in any case where antipyrin is contra-indicated. The antipyrin may be given in simple solution in water, and the antifebrin may be given either in pill or suspended in some mucilaginous mixture. Should neither antipyrin or antifebrin completely or promptly check the pain, resort must be had to morphia, unless the character of the patient is such that the morphine habit is to be feared, and even then it may often be given in such a shape that the patient is not aware of what is being taken. The form of morphia to be used will vary accordingly as the neuralgia is of the trigeminus, of the extremities, or of the abdomen. For abdominal neuralgia a combination of the sulphate of morphia with atropia is the best form, such as is combined in the hypodermic tablets put up by many houses and containing morphia sulph., gr. $\frac{1}{8}$; and atropia, gr. 1-200. For the neuralgia of the extremities and the trigeminus a solution should be made of the acetete of morphia, of such strength that each drop will contain gr. 1/6, and this will keep best when a small quantity of carbolic acid is added to it. Either form of morphia is best administered hypodermically, although this is not absolutely necessary, and a dose should be gr. $\frac{1}{8}$ to 1/6.

Quinine and galvanism, when properly administered, will relieve pain quite as effectively as these analgesics, but they will do it much more slowly and gradually, although often more effectively. An occasional dose of quinine, should be given at bed time, say 5 grains in capsule, and this should be continued for two or three nights and then stopped, only to be renewed occasionally when there is a relapse.

Galvanism is the most effective agent that we have for the final relief of neuralgia of the trunk and extremities, although it does not seem to have much effect upon neuralgia of the trigemus. To apply galvanism properly it is necessary to have a good battery, a measuring apparatus, and proper electrodes. There are a number of galvanic batteries to be had, but the most of them require to be filled so often as to be very inconstant in their current, and it is difficult at any time to know how much electricity is actually flowing. I consider the chloride of silver battery to be the best, most constant, and most portable that is made. A measuring instrument, graded into the electrical units which are known as milliamperes, and which is called a milliampere-meter, can now be had in the country of very excellent make, and is indispensable in determining the amount of current that is passing at any given time. It is impossible to give directions by indicating the number of cells, because the cells vary so much from day to day, accordingly as they have been filled or not, except in the case of the chloride of silver cells, which give forth a constant current for upwards of two years without need of repair; and even with this latter the varying conductivity of the skin in different individuals will allow a greater or lesser current to pass in one person than in another. Nor is the sensation of the patient any guide as to the amount of the electral current, because the sensation may be hightened or decreased in cases of neuralgia. We must therefore grade the current by one of these measuring instruments, one of the milliampere-meters, if galvanism is to be used with any precision. The electrodes should differ in size according to the part of the body to be galvaniszed. One pair should be about 5 x 6 inches, another one should be 4 x 2 inches, and a fourth one should be of round shape, of one inch diameter. These electrodes should be carefully covered with a fine, closely cut sponge, or with absorbent cotton, fastened with a small elastic band. Whenever they are applied, the skin should be well moistened with hot water, and the electrodes should also be thoroughly saturated in this, in order to diminish the non-conductivity of dry skin and dry sponge or cotton. Furthermore, in order to apply galvanism properly, it is necessary to study the so-called "motor points" which have been mapped out of late years by electricians, and a chart of which can be found in any of the electrical text-books. These motor points, as is well known, are spots where the nerve-trunks or muscular nerve-filaments are most superficial and most readily attacked by the electrical current. Turning on a current from ten cells, one of the larger electrodes should be placed upon the skin, moistened as above, at some distance from the motor point of the affected nerve, and then a smaller electrode, the one of three inches diameter, should be gradually placed upon this motor point. If the current then be unfelt by the patient, it should be gradually increased until about ten milliamperes are seen to pass. Even if not felt then, it should not be pushed further; but if the patient has a painful sensation in the affected nerve from a lesser current, the strength of the current should be diminished so as to give no pain at all. The best spot upon which to place the large electrode not used upon the motor point is the sternum. The current shonld be passed for about five minutes, and every day, unless it is thought that every other day will answer the purpose. After the motor point has thus been treated, the two large electrodes should be well wet with water, and one applied in the nape of the neck, whilst the other is put over the lower dorsal spine, the skin having first been well wet with hot water, and a current of ten to fifteen milliamperes passed for three times at the first sittings. In using these large electrodes, great care should be taken to first put one in place and then bring the other down upon the skin very gradually and carefully, so as to avoid any shock, and the same precautions should be used in taking them away. Often, too, it will be found very useful to apply one of the large electrodes at the upper part of the affected limb and the other at its lower part. The great advantage of galvanism is that it can be given in these doses for almost any length of time with perfect impunity, provided that care be taken in the manner indicated to avoid shocks from sudden interruptions of the flow of the current.

As the quinine and galvanism and the rest manifest their effect upon the neuralgia, the analgesics should be entirely removed, and at this stage of the treatment it will usually be found extremely useful, unless the individual be full-blooded and red-blooded, to make free use of iron, best of the dialized iron in one drachm doses, three times daily, after meals, in a half a teacup of water. In young individuals, in the colder months of the year, cod liver oil may also be of great use.—*Daniel's Texas Journal.*

